# New Protocetid Whale from the Middle Eocene of Pakistan: Birth on Land, Precocial Development, and Sexual Dimorphism

**DOI:** 10.1371/journal.pone.0004366

**Published:** 2009-02-04

**Authors:** Philip D. Gingerich, Munir ul-Haq, Wighart von Koenigswald, William J. Sanders, B. Holly Smith, Iyad S. Zalmout

**Affiliations:** 1 Museum of Paleontology and Department of Geological Sciences, University of Michigan, Ann Arbor, Michigan, United States of America; 2 Geological Survey of Pakistan, Quetta, Pakistan; 3 Steinmann-Institut für Geologie, Mineralogie und Paläontologie, Universität Bonn, Bonn, Germany; 4 Museum of Paleontology, University of Michigan, Ann Arbor, Michigan, United States of America; 5 Museum of Anthropology, University of Michigan, Ann Arbor, Michigan, United States of America; University of Chicago, United States of America

## Abstract

**Background:**

Protocetidae are middle Eocene (49–37 Ma) archaeocete predators ancestral to later whales. They are found in marine sedimentary rocks, but retain four legs and were not yet fully aquatic. Protocetids have been interpreted as amphibious, feeding in the sea but returning to land to rest.

**Methodology/Principal Findings:**

Two adult skeletons of a new 2.6 meter long protocetid, *Maiacetus inuus*, are described from the early middle Eocene Habib Rahi Formation of Pakistan. *M. inuus* differs from contemporary archaic whales in having a fused mandibular symphysis, distinctive astragalus bones in the ankle, and a less hind-limb dominated postcranial skeleton. One adult skeleton is female and bears the skull and partial skeleton of a single large near-term fetus. The fetal skeleton is positioned for head-first delivery, which typifies land mammals but not extant whales, evidence that birth took place on land. The fetal skeleton has permanent first molars well mineralized, which indicates precocial development at birth. Precocial development, with attendant size and mobility, were as critical for survival of a neonate at the land-sea interface in the Eocene as they are today. The second adult skeleton is the most complete known for a protocetid. The vertebral column, preserved in articulation, has 7 cervicals, 13 thoracics, 6 lumbars, 4 sacrals, and 21 caudals. All four limbs are preserved with hands and feet. This adult is 12% larger in linear dimensions than the female skeleton, on average, has canine teeth that are 20% larger, and is interpreted as male. Moderate sexual dimorphism indicates limited male-male competition during breeding, which in turn suggests little aggregation of food or shelter in the environment inhabited by protocetids.

**Conclusions/Significance:**

Discovery of a near-term fetus positioned for head-first delivery provides important evidence that early protocetid whales gave birth on land. This is consistent with skeletal morphology enabling *Maiacetus* to support its weight on land and corroborates previous ideas that protocetids were amphibious. Specimens this complete are virtual ‘Rosetta stones’ providing insight into functional capabilities and life history of extinct animals that cannot be gained any other way.

## Introduction

**Figure 1:**
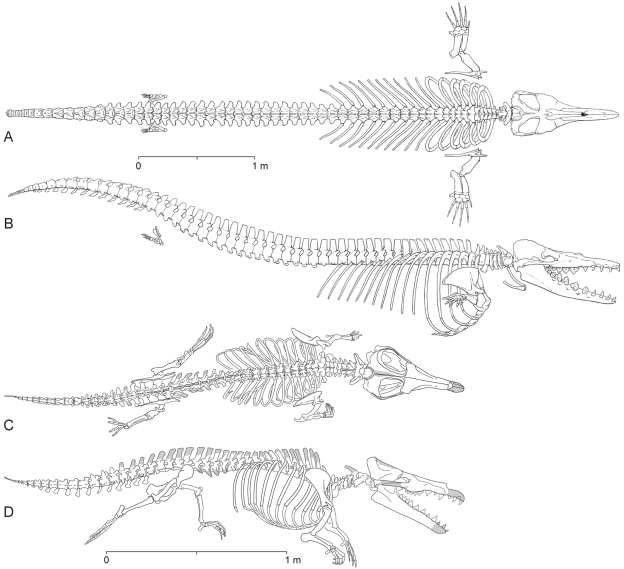
Skeletons of the Eocene archaeocete whales *Dorudon atrox* and *Maiacetus inuus* in swimming pose. (A, B)– *Dorudon atrox* (5.0 m; 36.5 Ma) based on UM 101222 and 101215 [11] in lateral and dorsal views, respectively. (C, D)– *Maiacetus inuus* (2.6 m; 47.5 Ma) based on male specimen GSP-UM 3551 in lateral and dorsal views, respectively.

Archaeoceti are basal cetaceans that document the evolutionary transition of whales from land to sea during the Eocene before the appearance of modern Mysticeti and Odontoceti [1]. Archaeocetes are primitive compared to later cetaceans in retaining cheek teeth with shearing crests, flexible elbow joints, and well-formed hind limbs with feet and toes (Figure 1). Skulls with long jaws, pointed incisors, and cuspate cheek teeth indicate that archaeocetes were specialized piscivores.

Two stages of archaeocete evolution, Protocetidae and Basilosauridae, are known from articulated skeletons. Protocetidae are middle Eocene in age (Lutetian-Bartonian, 49–37 Ma), and comprise fifteen genera and 16 species that range from South Asia and Africa to North America [2]. All are found in marine sedimentary rocks. The best known protocetids are 2 to 3-meter long primitive forms such as *Artiocetus* and *Rodhocetus* represented by skulls and partial skeletons with artiodactyl-like ankle bones [3]. These were amphibious foot-powered swimmers [4] that retained fully developed hind limbs connected to the vertebral column, enabling limb-supported locomotion on land. *Maiacetus*, described below, was part of this early diversification (Figure 1C, D). Basal archaeocetes have long been interpreted as amphibious, feeding in the sea but returning to land to rest, mate, and give birth [5], [6]. Late early Eocene and early middle Eocene pakicetid and ambulocetid archaeocetes, known from less complete remains, were almost certainly semiaquatic like protocetids [7], [8], [9].

Basilosauridae are late middle Eocene and late Eocene in age (Bartonian-Priabonian, 40–34 Ma) and were widely distributed in the world's oceans. The best known is the 5-meter long *Dorudon* (Figure 1A, B). Basilosauridae retained protocetid-like skulls, but their hind limbs were greatly reduced, making them fully aquatic. Basilosaurids had a powerful tail with a terminal fluke and were clearly tail-powered swimmers like modern whales [10], [11]. Generalized Eocene protocetid and basilosaurid Archaeoceti such as Maiacetus and Dorudon were on or near the main line of early whale evolution leading to later Mysticeti and Odontoceti, which appeared at the end of the Eocene or beginning of the Oligocene.

Two specimens of a new early middle Eocene (ca. 47.5 Ma) protocetid whale are described below, providing the first nearly complete skeleton of a protocetid whale, the first remains of a fetal skeleton of an archaeocete, and the first direct evidence for birth, life history, and sexual dimorphism in early archaeocetes— at a time when whales were still amphibious mammals spending some time on land and some time in the sea.

## Methods

### Field Methods

Fossil specimens described here were located during surface surveys in Pakistan in 2000 and 2004. These were found as articulated skeletons, and each specimen was separated into blocks of manageable size along cracks during excavation. The blocks were encased in plaster jackets and transported to the laboratory for preparation.

### Preparation

**Figure 2:**
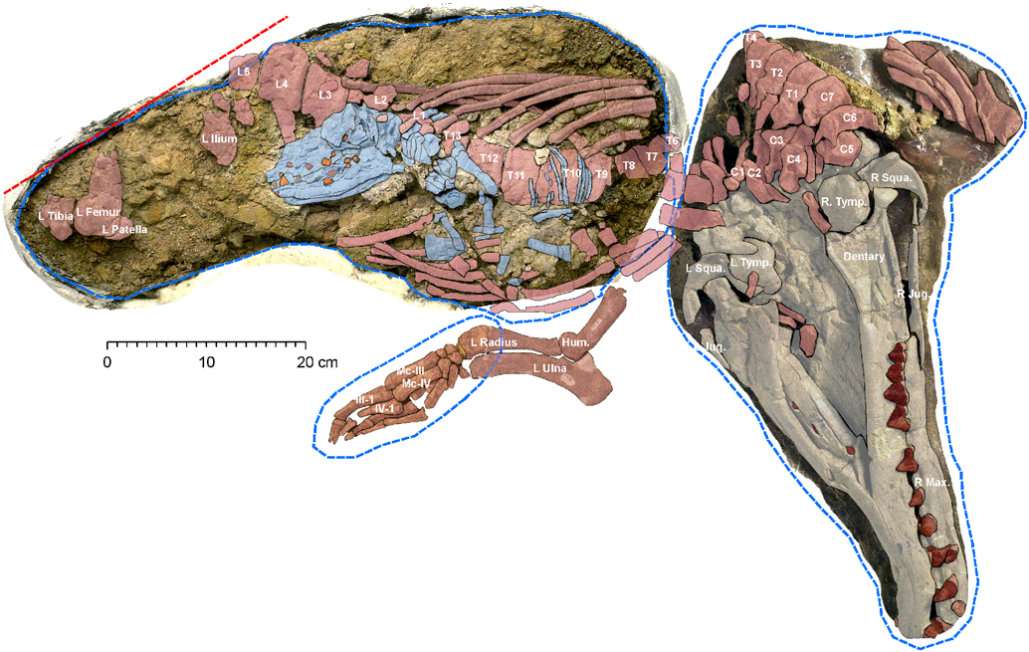
Adult female and fetal skeletons (type) of the protocetid *Maiacetus inuus*. Skull of the adult female (GSP-UM 3475a) is colored beige with brown teeth; her postcranial skeleton is colored red; the fetal skeleton (GSP-UM 3475b) is colored blue with red teeth. Blue dashed lines indicate the contours of the three field jackets and the red dashed line marks the edge of erosion.

The two specimens described here were prepared differently. The three plaster jackets of the adult female (GSP-UM 3475) were opened and cleaned to expose the articulated bones (Figure 2). The ventral surface of the cranium of the female whale was stabilized, supported by a fitted fiberglass cradle, and then turned and cleaned to expose the dorsal side of the cranium. Bones of the left forelimb were removed from the second jacket individually. The thorax and lumbus of the mother whale and the fetal skull and skeleton were stabilized in situ in the third jacket with only the side shown here being cleaned (Figure 2).

The nine plaster jackets of the adult male (GSP-UM 3551) were prepared manually with a micro-airscribe. Each block containing bones was molded and cast to create an archive of the position of the bones as found in the field. Each block was then fully prepared to free individual skeletal elements. These associated elements were then assembled into a full skeletal mount for exhibition in the University of Michigan Exhibit Museum (see Figure 1A, B).

### Imaging

The fetal dentition described here was imaged with computed tomography using a Philips Tomoscan AVE1 scanner at the Radiologische Klinik, Universität Bonn (Germany).

### Terminology

**Ma**, geological age in millions of years before present.

#### Anatomical Abbreviations in Text

*C*, cervical vertebra; *Ca*, caudal vertebra; *L*, lumbar vertebra; *Mc*, metacarpal; *Mt*, metatarsal; *R*, rib; *S*, sacral vertebra; and *T*, thoracic vertebra.

Teeth are numbered sequentially from front to back (mesial to distal), with upper and lower incisors (*I*), canines (*C*), premolars (*P*), and molars (*M*) distinguished by superscripts and subscripts, respectively.

#### Institutional Abbreviations

**GSP-UM**, Geological Survey of Pakistan-University of Michigan collection, housed in Quetta, provincial capital of Balochistan; **UM**, University of Michigan Museum of Paleontology, Ann Arbor, Michigan, United States of America.

## Results

### Systematic Paleontology

Mammalia Linnaeus, 1758

Cetacea Brisson, 1762

Archaeoceti Flower, 1883

Protocetidae Stromer, 1908

Protocetinae Stromer, 1908

***Maiacetus*** **gen. nov.**

#### Etymology

*Maia*, mother, and *ketos*, whale (Greek): named for the sex and gravid state of the holotype.

#### Type Species

*Maiacetus inuus* sp. nov.

#### Diagnosis

Medium-sized protocetid archaeocete with a skeleton 2.6 m in length and an estimated weight of 280–390 kg. Skull has the medium-length rostrum, anteriorly-positioned nares, large mandibular canals, narrow frontal shield, broad cranial base, and large tympanic bullae typical of early protocetids. Differs from contemporary *Artiocetus clavis* and *Rodhocetus balochistanensis* in having solidly co-ossified left and right dentaries. In the ankle, the astragalus has deep proximal and distal trochleae as in *Rodhocetus*, but differs in having an indented dorsolateral border; cuboid is less deeply notched for the calcaneum than in *Artiocetus* and more deeply notched than in *Rodhocetus*. Metacarpals, carpal phalanges, and all hind limb elements are shorter relative anterior thoracic centrum length than comparable ratios in *Rodhocetus*.

***Maiacetus inuus*** **sp. nov.**

Figures 1C–D, 5–9, 10C, 11B

#### Etymology

*Inuus*, god of fecundity (Latin): named to acknowledge both the exceptional recovery of a gravid female in the cetacean fossil record, and the importance of life history in mammalian evolution.

#### Holotype

GSP-UM 3475a, articulated skull, thorax, and left forelimb of an adult female. Holotype contains the skull and partially ossified skeleton of a near-term fetus, GSP-UM 3475b (Figure 2). The mother is a young adult as shown by the complete eruption of the permanent dentition, limited tooth wear, fusion of most epiphyses, and presence of a fetus.

#### Type Locality

**Figure 3:**
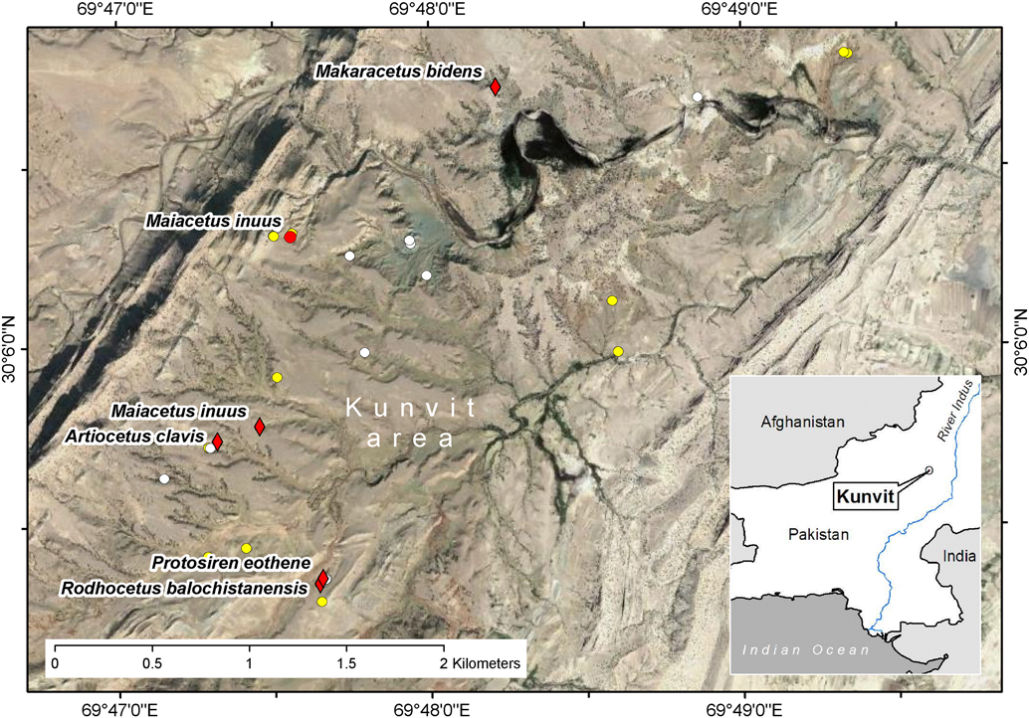
Map showing localities of some Eocene whales in eastern Balochistan (Pakistan). Map showing the Kunvit area in the southwestern part of Lakha Kach syncline, northwest of the town of Rakhni. Red diamonds mark type localities for *Artiocetus clavis* (GSP-UM 3458), *Rodhocetus balochistanensis* (GSP-UM 3485) [3], *Protosiren eothene* (GSP-UM 3487) [49], *Makaracetus bidens* (GSP-UM 3570; from younger beds of the overlying Domanda Formation) [2], and *Maiacetus inuus* (GSP-UM 3475a, b, female, fetal skeleton). Localities are in the upper part of the Habib Rahi Formation of early Lutetian age [12]. The red circle marks the locality of the referred specimen of *Maiacetus inuus* (GSP 3551, male), yellow circles show localities of other archaeocete specimens, and white circles show localities of other vertebrate specimens.

Kunvit, Kohlu District, eastern Balochistan Province, Pakistan (Figure 3). GPS coordinates are 30.0963°N and 69.7908°E (WGS84 datum).

#### Referred Specimen

GSP-UM 3551, virtually complete skeleton interpreted as male (see below; Figures 1C, D, 9). Found in Kunvit, Kohlu District, eastern Balochistan Province, Pakistan in the same strata and about 1 km from the holotype (Figure 3). GPS coordinates are 30.1051°N and 69.7926°E (WGS84 datum).

#### Age

**Figure 4:**
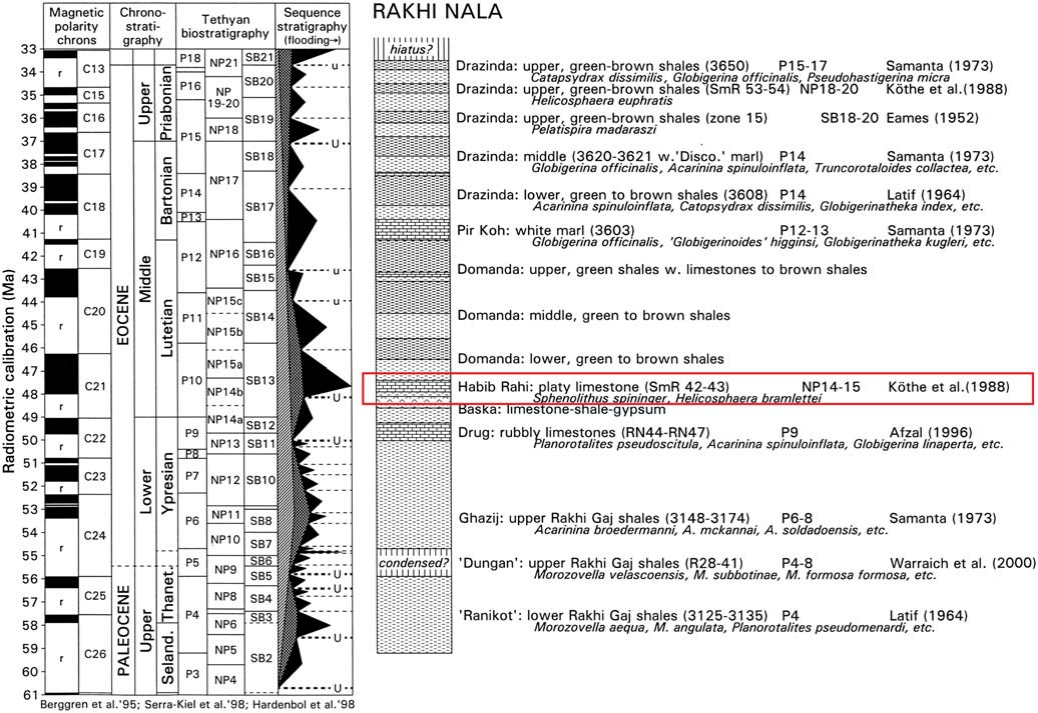
Temporal constraints on the age of *Maiacetus inuus*. Red rectangle highlights the platy limestones and marls of the upper part of the marine Habib Rahi Formation. Nannoplankton *Sphenolithus spininger* and *Helicosphaera bramlettei* indicative of NP zones 14–15 have been reported from the Habib Rahi Formation [13]. The age of *Maiacetus inuus* is about 47.5 Ma. Figure from [14].

All specimens are from upper beds of the Habib Rahi Formation, a succession of interbedded marine marls and shales. The marls are approximately 10–20 cm thick, and the shales are approximately 1 meter thick. The marls thin and the shales thicken toward the top of the section [12]. The age of the Habib Rahi Formation has been established on the east side of the Sulaiman Range at Rakhi Nala 35 km southeast of Kunvit [13], [14]. The specimens described here were found near the top of the major flooding sequence in the early Lutetian stage of the middle Eocene, calibrated to approximately 47.5 Ma (Figure 4).

#### Diagnosis

As for the genus.

### Description of *Maiacetus inuus*

#### Adult Female Skeleton (GSP-UM 3475a)

The adult female skeleton (GSP-UM 3475a) and its associated fetal skeleton (GSP-UM 3475b) were collected in three blocks: (1) a cranial block with the female skull, cervical vertebrae, some anterior thoracic vertebrae, and partial scapulae; (2) a block containing the left forelimb of the adult female; and (3) a block containing the thorax of the adult mother whale, and the skull and remaining skeleton of the fetus (Figure 2). Blocks 2 and 3 were slightly displaced (3–5 cm) relative to block 1 along a small fault or joint surface after the specimen was buried and fossilized. This movement deformed immediately adjacent bones and may have destroyed parts of bones near the fault. Some bone pieces removed from the fault zone during collection fit onto bones of one block or the other.

The adult female skull and skeleton came to rest on their dorsal surfaces before burial, as is typical for archaeocetes. This stomach-up orientation may be caused by a buildup of gases in the abdomen during decomposition. Skulls are seemingly more stable lying on their dorsal surface than in any other position.

**Figure 5:**
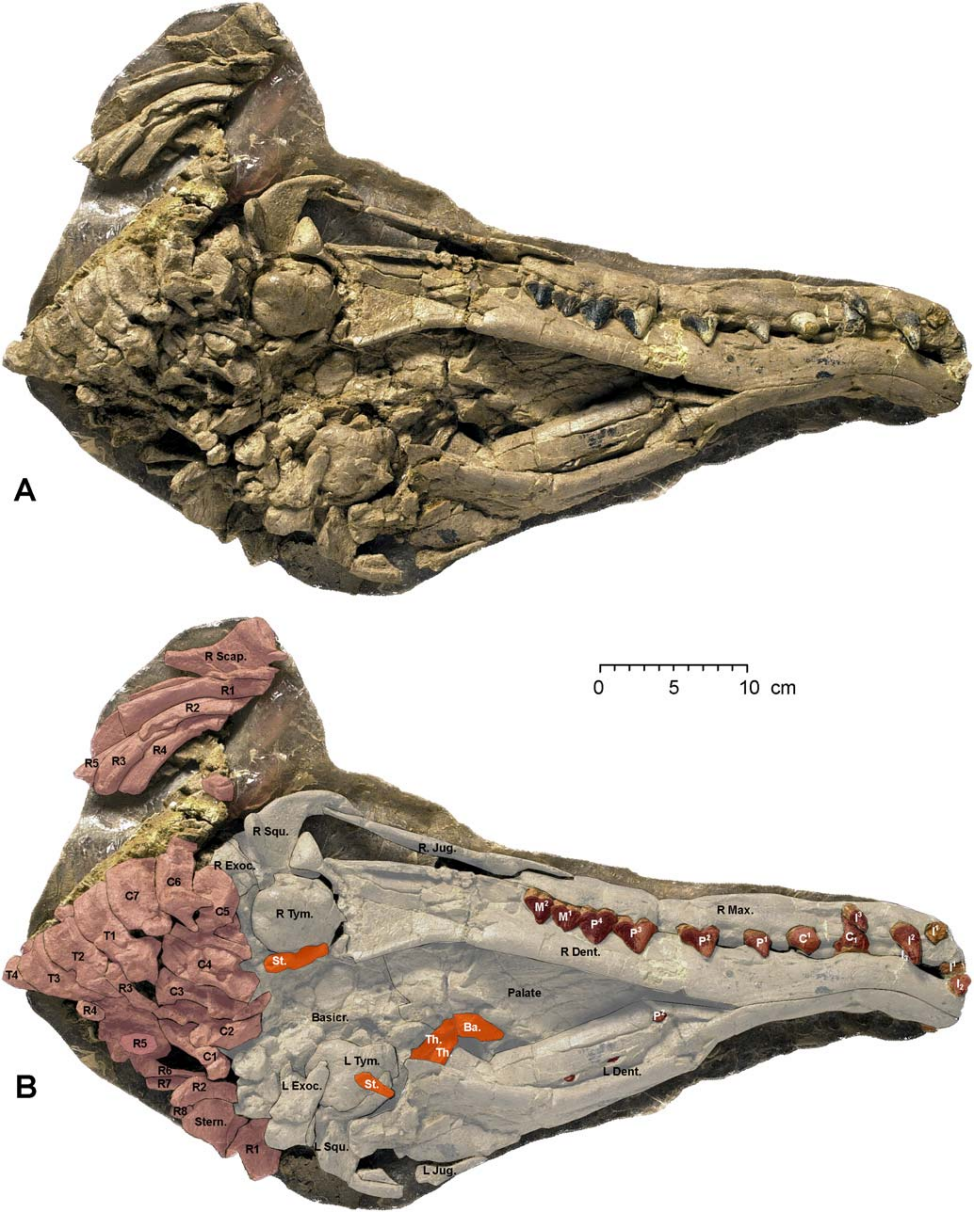
Skull of *Maiacetus inuus* (GSP-UM 3475a; female). Skull in ventral view (A) with interpretive shading and labels (B). Shaded components include skull bones (grey), teeth (brown), hyoids (orange), and postcranial bones (red). *Osteological abbreviations*: *Ba.*, basihyal; *Basicr.*, basicranium; *C*, cervical vertebra; *Dent.*, dentary; *Exoc.*, exoccipital; *Jug.*, jugal; *L*, left; *Max.*, maxilla; *R*, right, rib; *Scap.*, scapula; *Squ.*, squamosal; *St.*, stylohyal; *Stern.*, sternebrum; *T*, thoracic vertebra; *Th.*, thyrohyal; *Tym.*, tympanic.

**Figure 6 pone-0004366-g006:**
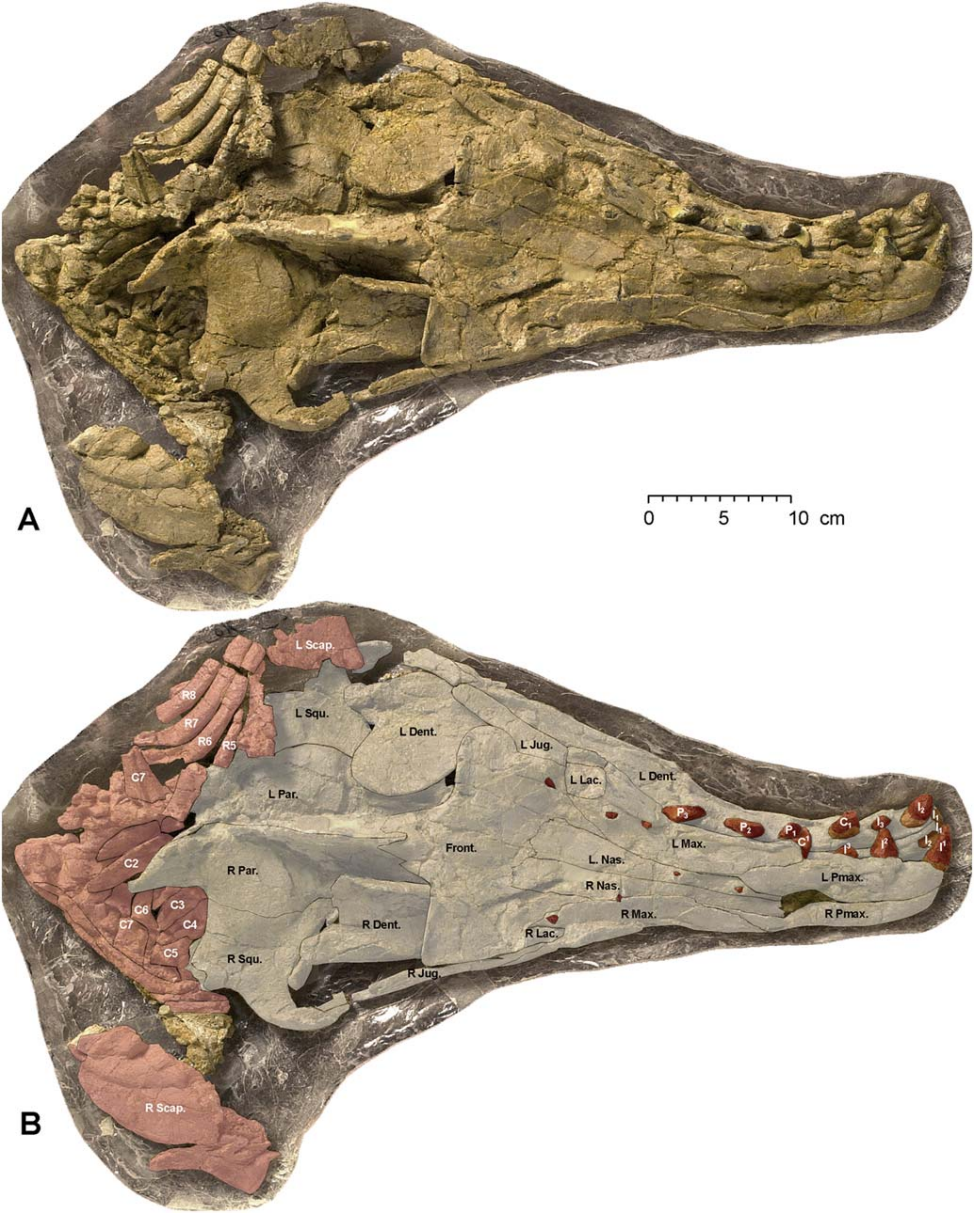
Skull of *Maiacetus inuus* (GSP-UM 3475a; female). Skull in dorsal view (A) with interpretive shading and labels (B). Shaded components include skull bones (grey), teeth (brown), and postcranial bones (red). *Osteological abbreviations*: *Dent.*, dentary; *Front.*, frontal; *Jug.*, jugal; *L*, left; *Lac.*, lacrimal; *Max.*, maxilla; *Nas.*, nasal; *Par.*, parietal; *Pmax.*, premaxilla; R, right, rib; *Scap.*, scapula; *Squ.*, squamosal.

The nearly complete skull of the adult female has been exposed in ventral and dorsal views (Figures 5, 6). It measures 56 cm in length (Table 1). Salient features of the skull include a relatively narrow and anteroposteriorly-elongated rostrum, with premaxillae expanded anteriorly in front of the canine teeth. The rostrum widens posteriorly and is relatively broad at the position of the left and right upper molars.

**Table 1:** Skeletal measurements (cm) of *Maiacetus inuus* compared to those of the previously known protocetids *Artiocetus clavis* and *Rodhocetus balochistanensis*, which are also from the early middle Eocene of Pakistan [3].

Measurement	*Artiocetus clavis*	*Rodhocetus balochistan-ensis*	*Maiacetus inuus*	*Maiacetus inuus*	*Maiacetus inuus*
	GSP-UM 3458 (Type)	GSP-UM 3485 (Type)	GSP-UM 3475a (Type, female)	GSP-UM 3551 (Male)	Male/female Ratio^2^
Cranial length	57.5		56.0		
Cranial width	44.0		27.0		
Tympanic bulla length	6.5		6.1	6.6	**1.09**
Tympanic bulla width	4.8		4.9	5.4	**1.12**
Mandibular foramen height	6.0			7.0	
Cervical vert. C4 length	2.8	3.2		2.7	
Thoracic vert. T4 length	3.2	3.9	2.7	4.0	**1.51**
Lumbar vert. L4 length	4.3		4.5	4.7	**1.04**
Caudal vert. C4 length		5.5		5.0	
Scapula length	20.0*			21.7	
Humerus length		21.0*		21.5	
Ulna length			14.6	17.5	**1.20**
Ulna olecranon length			4.6	5.7	**1.24**
Ulna prox. articular breadth			1.9	2.0	**1.05**
Radius length		11.0	10.0	11.7	**1.17**
Radius distal width	2.7	3.0		2.5	
Radius distal thickness	2.3	1.8		2.3	
Carpus length		2.3	2.1	2.2	**1.05**
Pisiform length		4.7		3.7	
Metacarpal length (Mc-III)		7.8	6.1	6.5	**1.07**
Manual phalanx length (III-1)		6.3	5.9	5.9	**1.00**
Manual phalanx length (III-2)		3.3*	2.6	3.8*	**1.48**
Manual phalanx length (III-3)		1.3*	1.1	1.4	**1.26**
Ilium length (acetabular margin)	12.4			12.4	
Acetabulum diameter	2.8*	3.1*		3.1	
Femur length		19.0		17.2	
Femur midshaft diameter (anteroposterior)	2.2	2.3		2.0	
Femur midshaft diameter (bilateral)	2.5	3.2		2.5	
Patella length	3.1	4.1		2.9	
Patella width	1.9	2.6		1.8*	
Patella thickness	1.9	2.3		1.6	
Tibia length		21.0		18.6	
Astragalus length	4.4	5.4		3.8	
Astragalus width	2.1	2.3		2.0	
Astragalus height	2.1	2.9		2.2	
Cuboid length	1.9	2.7		1.9	
Metatarsus length (Mt-III)		12.2		8.9	
Pedal phalanx length (III-1)		9.0		7.0	
Pedal phalanx length (III-2)		7.0		4.7	
Pedal phalanx length (III-3)		2.3		1.8	
Median dimorphism					**1.12**
1Body weight (kg; estimate)	400*	410*	280*	390*	**1.39** *

Sexes of the *A. clavis* and *R. balochistanensis* specimens are not known.

* Asterisk indicates estimated measurement.

1 Body weight is estimated from the centrum size of available thoracic vertebrae, using multiple regression and a reference set of 13 marine mammal skeletons of known weight (8 cetaceans and 5 pinnipeds) spanning a range of 85 to 23,500 kg (see Figure 13). Weight for female *Maiacetus inuus* is estimated from male *M. inuus* in two ways: using the 12% average difference in comparable linear dimensions or the 4% average difference found for premolars and molars (Table 2).

2 Ratio based on measurements of male and female adult specimens in *Maiacetus inuus*.

On the dorsal surface of the cranium, the external nares open above the upper canine teeth (Figure 6). The nasals are narrow and extend posteriorly to overlap the frontals. Expanded frontals form a frontal shield with prominent supraorbital processes. The frontal shield is not as broad relative to the rest of the skull as it is in later protocetids. The jugals are relatively thin and flare posteriorly. The skull is broadest across the left and right squamosals. There is a low sagittal crest and a moderately developed nuchal crest.

On the ventral surface of the cranium the glenoid fossa for articulation of the dentary is open and relatively flat as in other early protocetids. Remnants of the basihyal, tympanohyals, and stylohyal bones can be identified on the ventral surface of the basicranium (Figure 5). The tympanic bullae are large with a sigmoid process well developed laterally and involucrum well developed medially.

The mandibular symphysis is solidly fused from the anterior end of the dentaries to a position beneath P_2_ (Figure 5). The ventral portions of the dentaries are hollow posteriorly, and the mandibular canal is well developed. The canal cannot be measured, however, due to dorsoventral compression of the dentaries.

Dorsoventral compression of the adult female skull has pushed some of the crowns of lower teeth into the bones of the cranium and some crowns of upper teeth into the left dentary (Figures 5, 6). The upper dental formula is 3.1.4.3 (incisors, canines, premolars, molars). Partial or complete crowns of all upper teeth are exposed in the right premaxilla and maxilla (Figure 5), except for M^3^, which is known in the adult male (GSP-UM 3551; see below). All incisors are caniniform with simple conical crowns on a single root. I^1–2^ are large and I^3^ is relatively small (Table 2). The canine is moderate in size and simple in form, with a conical crown on a single root. P^1^ has a simple crown and is single-rooted, while P^2–4^ have more elongated crowns and double roots. Upper molars have a simple large external paracone, with little development of a metacone. Protocones on upper molars are not exposed in the adult female skull but are well developed in the adult male (GSP-UM 3551; see below). P^3^ has the anteroposteriorly longest crown in the upper dentition (Figure 5).

**Table 2 pone-0004366-t002:** Measurements (mm) of teeth in female and male specimens of *Maiacetus inuus*.

Tooth pos.	Upper teeth				Dimorphism	Tooth pos.	Lower teeth				Dimorphism^1^	All
	Female GSP-UM 3475a		Male GSP-UM 3551				Female GSP-UM 3475a		Male GSP-UM 3551			Median M/F dimorphism
	Left	Right	Left	Right	M/F		Left	Right	Left	Right	M/F	
**I^1^** L	16.84					**I_1_** L						
W	12.28					W						
H	29.07					H						
**I^2^** L	18.26	18.17				**I_2_** L	18.00*	17.80				
W		12.61				W		16.30				
H	23.49	29.03				H						
**I^3^** L	16.01	14.91				**I_3_** L	14.17					
W		9.36				W	9.52*					
H	12.60	15.24				H	12.43*					
**C^1^** L		19.23		23.80*	**1.24**	**CI_1_** L	18.09					
W	13.21*			13.65	**1.03**	W	10.95					1.20
H	26.00			31.30	**1.20**	H						
**P^1^** L		16.81		16.24	**0.97**	**P_1_** L	15.69					
W		7.76		8.98	**1.16**	W	8.69					
H		16.13		17.06	**1.06**	H	14.10					
**P^2^** L		28.60		29.00	**1.01**	**P_2_** L	28.28		28.97		1.02	
W				12.72		W	11.00*		11.44		1.04	
H				27.25		H			27.64			
**P^3^** L		28.65		33.79	**1.18**	**P_3_** L						
W				16.40		W	11.60		12.06		1.04	
H				25.04		H			30.42			
**P^4^** L		25.46		25.57	**1.00**	**P_4_** L			33.15			
W				20.22		W			12.20			1.04
H				23.68		H			30.33			
**M^1^** L		19.93		20.21	**1.01**	**M_1_** L			22.35			
W						W			9.74			
H						H						
**M^2^** L		20.00		21.39	**1.07**	**M_2_** L			25.85			
W				17.40		W			12.50			
H				15.99		H			26.32			
**M^3^** L				14.00		**M_3_** L			24.26			
W				12.16		W			12.61			
H				15.22		H			23.48			

* Asterisk indicates estimated measurement.

1 Ratios quantifying male-to-female tooth size dimorphism are provided when teeth at the same position can be compared. Median dimorphism in canine size, based on upper canines, is 1.20, meaning males have canines on the order of 20% larger than those of females. Median dimorphism in premolar and molar size is 1.04, meaning that males have premolars and molars only about 4% larger than those of females.

The lower dental formula, like the upper, is 3.1.4.3. I_1–3_, C_1_, and P_1–3_ are present in the left dentary (Figure 6), and the remaining premolar and lower molars are known in the adult male (GSP-UM 3551; see below). Again, all incisors are caniniform with simple conical crowns on a single root. I_1_ is relatively small (judging from the root), I_2_ is large, and I_3_ is small (Figure 6, Table 2). Lower canine C_1_ is moderate in size and simple in form, with a conical crown on a single root. Lower P_1_ is small, and P_2–3_ have anteroposteriorly longer crowns. The longest tooth in the lower dentition was probably P_3_, but this cannot be determined with confidence from the specimens at hand.

The cervical vertebrae (C1–C7) of the adult female (GSP-UM 3475a) are preserved in articulation in the cranial block, but these are difficult to study or measure individually (Figures 2, 5). The first three thoracic vertebrae and a remnant of the fourth (T1–T4) are preserved in articulation posterior to the cervicals. Nothing has been recovered of T5, which was evidently lost during displacement of cranial and thoracic blocks along the intervening fault or joint surface. The vertebral column continues in the thoracic block. T6 is represented by a piece of the centrum, while the remaining thoracic vertebrae (T7–13) and lumbar vertebrae (L1–L4 and part of L5) are preserved in articulation (Figure 2).

A sternebrum and some ribs are present in the cranial block (Figures 5, 6). The ribs continue into the thoracic block, where they are preserved for the most part in order. The distal ends of the right ribs lie on top of the vertebral centra, whereas distal ends of the left ribs are displaced more laterally. Ribs in the thoracic block can only be identified by counting backward from the most distal rib (R13).

**Figure 7 pone-0004366-g007:**
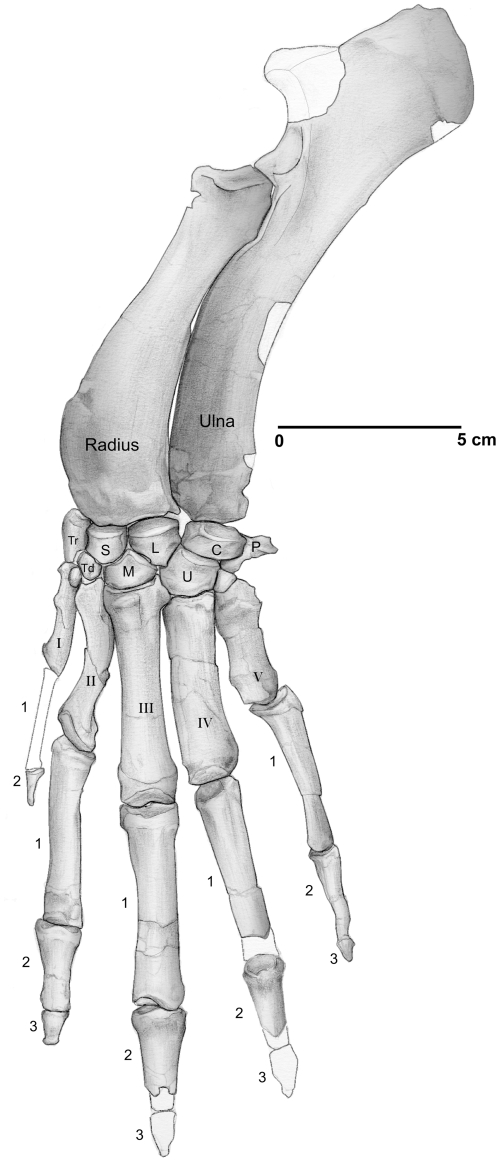
Left radius, ulna, carpus and manus of *Maiacetus inuus* (GSP-UM 3475a, female). Open areas are reconstructed. *Abbreviations*: *C*, cuneiform; *L*, lunar; *M*, magnum; *P*, pisiform; *S*, scaphoid; *Tr*, trapezium; *Trd*, trapezoid; *U*, unciform.

The left forelimb was preserved in articulation on the left side of the thorax (Figures 2, 7). A fragment of the left scapula lies under the left squamosal, and a fragment of the right scapula lies under the anterior ribs on the right side of the cranium. Much of the left scapula and the proximal end of the left humerus were damaged at the juncture of the cranial and forelimb blocks. The distal end of the humerus is relatively narrow with a shallow trochlea and deep olecranon fossa for articulation with the ulna. The radius and ulna are well preserved although somewhat flattened (Figure 7).

The distal row of carpal bones is alternating rather than serial relative to the proximal row. Metacarpal I is the smallest, metacarpals III and IV are the largest, and metacarpal III is distinctly longer than metacarpal IV. With the exception of the pollex (digit I), all of the proximal phalanges are about the same length (the proximal phalanx of digit I is not known). Similarly, middle phalanges of digits II through V are similar in length, although phalanx III-2 is distinctly more robust. Terminal phalanges of digits I and V are simple and pointed and probably did not bear a hoof, whereas the blunt end of terminal phalanx II-3 indicates that it was hoof bearing. Terminal phalanges II-3, III-3, and IV-3 in the adult male (GSP-UM 3551) all bore hooves.

Preservation of the vertebral column ends at L5. Nothing remains of L6, the sacrum, or the tail. Very little of a hind limb is preserved in the adult female (Figure 2). The left ilium is represented only by the crescent-shaped anterior margin. The distal left femur, patella, and proximal end of the left tibia are preserved in articulation, but the rest of the left hind limb and all of the right hind limb are missing.

#### Fetal Skeleton within Adult Female (GSP-UM 3475b)

The fetal skeleton (GSP-UM 3475b) is preserved within the ribcage of the type specimen (GSP-UM 3475a) (Figure 2, colored blue). This immature individual is interpreted as a fetal skeleton, rather than an ingested meal, because of the absence of any damage to the skull. Protocetids had shearing molars used to slice and chew their prey. The skull of the fetal skeleton could not have survived such mastication and be as well preserved as it is.

The fetal skeleton preserves the skull, some cervical and thoracic vertebrae, ribs, and portions of the fore- and hind limbs. The skull includes the cranium and left and right lower jaws, with crowns and roots of many deciduous teeth in place in the upper and lower jaws. The surfaces of the fetal bones are porous in contrast to the smoother surfaces of adult bones. The fetal skeleton has a preserved length of about 33 cm, and probably had a total length approximately twice the preserved length.

**Figure 8 pone-0004366-g008:**
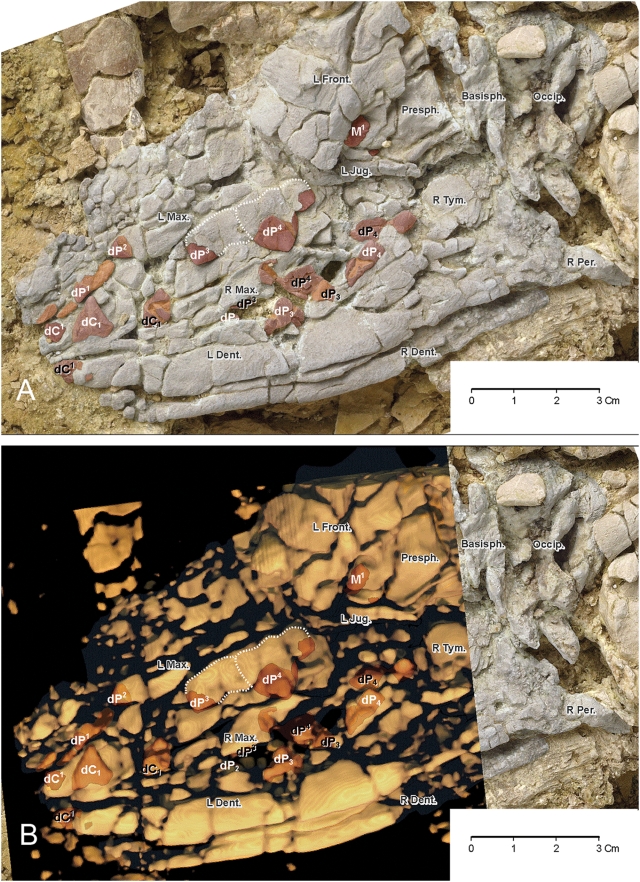
Fetal skull of *Maiacetus inuus* (GSP-UM 3475b). (A)–Photograph showing bones (shaded white) and teeth (shaded brown) in lateral view. (B)–CT image overlain on tooth-bearing part of fetal skull in lateral veiw. *Abbreviations*: left frontal (*L. Front.*), left jugal (*L Jug.*), occipital (*Occip*.) with left and right condyles flanking the foramen magnum, and left and right dentaries (*L dent.*, *R dent.*). Teeth are shaded brown, with darker brown representing enamel, and lighter brown representing roots and exposed dentine. White and black labels identify teeth of the left and right sides, respectively. Dotted lines trace outlines of fully formed crowns of left dP^3^ and dP^4^. These crowns are visible on the surface (A) where thin bone of the maxilla is pressed over more rigid underlying crowns, and as denser masses in the CT scan (B). Remaining teeth are identified by size and position relative to dP^3^ and dP^4^. Note presence of the developing crown of permanent left M^1^ posterodorsal to the crown of left dP^4^ (dorsal to the left jugal and posterior to the left frontal). Partial crown of right M^1^ (unlabeled) is visible just below and posterior to left M^1^.

The length of the fetal skull, as preserved, is 15 cm, and this probably measured 17 cm when the missing anterior end of the rostrum is added (Figure 8). Identifiable bones include the left frontal, the left jugal, the occipital with left and right condyles flanking the foramen magnum, and the left and right dentaries.

The fetal dentition is largely intact but interpretation is complicated by bilateral compression of the skull (Figure 8A, B). Left upper teeth are displaced dorsally relative to right upper teeth, and the left dentary is displaced slightly anteriorly relative to the right dentary. Teeth from both left and right sides, being denser and harder than surrounding fetal bone, all show on the exposed left side of the skull.

The largest tooth in each quadrant is the distinctive fourth deciduous premolar. These four premolars (left and right dP^4^ and dP_4_), serve as landmarks for identification of more anterior and posterior teeth. Anterior to dP^4^ and dP_4_, there are partial crowns of all of the third deciduous premolars (left and right dP^3^ and dP_3_), as well as upper and lower deciduous canines. Teeth anterior to dP^3^ and dP_3_ were identified by their morphology and by their stage of development, using an undescribed protocetid skull with a good deciduous dentition for comparison.

Posterior to dP^4^, there are partial crowns of the left and right first permanent upper molars visible below the left frontal and above the left jugal (Figure 8,). Left M^1^ is the most clearly exposed and it appears to have the main paracone cusp almost fully formed, with the crown as a whole about one-half mineralized. Crowns of left and right lower permanent molars are not visible but were presumably also partially mineralized.

Cervical and anterior thoracic vertebrae are compressed in articulation, and the ribs are preserved as narrow ribbons of porous bone pressed onto the ventral surfaces of the mother's thoracic vertebrae. Parts of seven long bones are evident. The largest of these are presumably humeri, and there may be remnants of ulnae and radii as well. Seven long bones would exceed the number expected in a pair of protocetid forelimbs, and so some may pertain to the hind limbs. More precise identification is hindered by the absence of ossified epiphyses.

#### Adult Male Skeleton (GSP-UM 3551)

**Figure 9:**
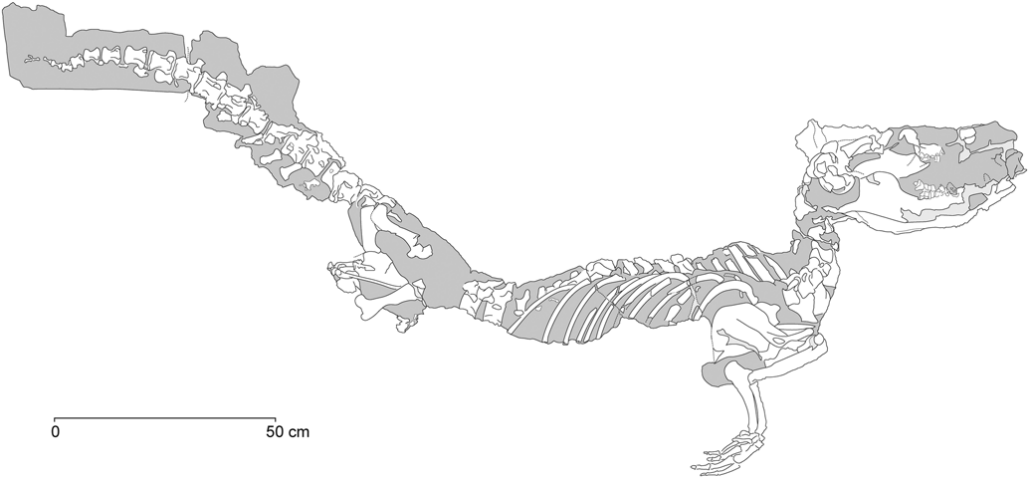
Skeleton of *Maiacetus inuus* (GSP-UM 3551, male). Skeleton as preserved in left lateral view. Gray shading is marl matrix (see Figure 1C, D for skeletal restoration).

The most complete skeleton of *Maiacetus inuus* is that of a second adult (Figure 1C, D) found in articulation in the field (Figure 9). The skull lacks the anterior portion of the rostrum, while the vertebral column and fore- and hind limbs are virtually complete. The specimen is interpreted as male because it is larger than the female skeleton described above, the canine teeth are larger relative to the size of other teeth, and the pelvic morphology is male (see below). Comparative measurements are provided in Tables 1–3.

**Table 3:** Vertebral centrum measurements (mm) for adult male *Maiacetus inuus* (GSP-UM 3551; Figure 9).

Vertebra	Length	Width	Height	Vertebra	Length	Width	Height
**C1**	23.5	89.5	63.3	**S1**	47.8	60.4	34.6
**C2**	34.5	37.7	34.5	**S2**	44.7	58.4	32.8
**C3**	28.3	42.5	34.0	**S3**	48.5	54.1	34.4
**C4**	26.6	39.3	31.9	**S4**	52.2	51.8	37.5
**C5**	25.4	37.9	31.7	**Ca1**	47.6	52.7	42.9
**C6**	28.6	41.4	32.6	**Ca2**	47.5	51.6*	41.7
**C7**	27.7	49.9	29.5	**Ca3**	49.4	—	47.9
**T1**	34.4	48.6	35.2	**Ca4**	50.2	—	48.8
**T2**	35.8	50.8	35.4	**Ca5**	52.8	—	(49.4)
**T3**	35.8	52.9	35.7	**Ca6**	54.2	—	(50.5)
**T4**	40.1	52.7	40.0	**Ca7**	59.8	—	(49.4)
**T5**	35.0	54.7	39.5	**Ca8**	61.1	—	(48.8)
**T6**	36.0	56.2	36.4	**Ca9**	60.0	—	(47.0)
**T7**	36.4	56.8	(36.7)	**Ca10**	60.0	—	(44.0)
**T8**	36.5	57.1	(37.0)	**Ca11**	57.4	—	(41.0)
**T9**	36.4	58.0	(37.3)	**Ca12**	51.9	—	(36.5)
**T10**	35.9	59.6	37.7	**Ca13**	45.2	27.0*	31.0*
**T11**	39.9	58.0	36.8*	**Ca14**	37.6	29.0	27.8
**T12**	39.7	54.0	(38.0)	**Ca15**	29.7	24.7	16.3
**T13**	39.5	52.4	39.2	**Ca16**	23.9	19.5	11.4
**L1**	40.1	51.9	39.6	**Ca17**	24.7	14.2	9.2
**L2**	41.2	48.6	(41.5)	**Ca18**	20.3	8.6	5.1
**L3**	46.0	54.3	42.2	**Ca19**	19.9	6.6	3.8
**L4**	46.5	60.2	40.0	**Ca20**	16.7	6.6	5.0
**L5**	46.1	65.3	40.2	**Ca21**	11.1	4.8	3.1
**L6**	52.2	65.1	37.8				

*Abbreviations*: *C*, cervical vertebra; *Ca*, caudal vertebra; *L*, lumbar vertebra; *S*, sacral vertebra; *T*, thoracic vertebra.

* Asterisk indicates estimated measurement.

( ) indicate interpolated measurement.

— Ca3 through Ca12 are bilaterally compressed, precluding measurement of their width.

The male skull is similar to that of the female described above. Here again the anterior rostrum is narrow but it expands rapidly at mid length toward the posterior end of the skull. The palate is much broader posteriorly than it is anteriorly. The frontal shield is expanded with broad supraorbital processes, but it is again narrower than that of later protocetids. Lacrimal bones are exposed on the face anterior to the orbits. The high, narrow, and deep nuchal area is bordered ventrolaterally by robust, broadly-spaced parocciptal processes, suggesting the presence of powerful neck muscles. Tympanic bullae are large, robust, and firmly attached to the underside of the endocranium. The convex occipital condyles are oriented posteriorly.

The mandibular symphysis is fused as far posteriorly as the distal margin of P_2_. The large mandibular foramen leading to the mandibular canal is mediolaterally narrow and dorsoventrally deep (depth estimated as 7 cm). It opens internally at the posterior end of the dentary. The mandibular condyles are positioned well below the apex of the coronoid process, and the gonial angle of the mandible is compressed and square in outline (viewed laterally; Figure 1A).

Upper and lower incisors are missing in the adult male (GSP-UM 3551), but the upper canine (C^1^) is large and anteroposteriorly expanded. The upper first premolar (P^1^) is small, while P^2^ is closer in size to P^3^ and P^4^. P^3^ has the largest crown of the upper cheek teeth. P^3^-M^3^ are closely spaced with no diastemata. A protocone is well developed on P^4^ and on M^1^. This primitive cusp decreases in size on M^2^ and nearly disappears on M^3^. In the lower dentition, C_1_ and P_1_ are represented only by impressions of the crowns and roots. The crown of the lower canine crown is longer anteroposteriorly and the root thicker than in the female (GSP-UM 3475a), while P_1_ is small. Lower P_2_, P_3_, and P_4_ are all relatively large, with P_3_ or P_4_ the largest of the lower cheek teeth. The lower molars are closely spaced, with a high anterior cusp (protoconid) and lower posterior cusp (hypoconid).

The vertebral formula (cervicals, thoracics, lumbars, sacrals, caudals) is 7C-13T-6L-4S-21Ca for a total of 51 vertebrae, which differs by only two caudal vertebrae from the formula found in some primitive artiodactyls [15]. Precaudal vertebrae increase in size along the series as in other protocetids (Table 3). The atlas (C1) has broad transverse processes, moderately deep and curved prezygapophyses, and a complex foraminal pathway for the vertebral arteries to accommodate motion of the skull. In C3–C7 the orientation of zygapophyses, the saddle-shape of vertebral body surfaces, and the imbrication of transverse processes suggest that the neck was more capable of dorsiflexion than lateral rotation. Anterior thoracic vertebrae have long spinous processes for muscles and ligaments inserting on the exoccipital region and nuchal area of the skull to stabilize the head. Thoracic T11, the diaphragmatic vertebra, has thoracic-like prezygapophyses and lumbar-like postzygapophyses. T12 is the anticlinal vertebra with a vertical neural spine (Figure 1A). In the lumbus, anterodorsally inclined spinous processes, non-revolute zygapophyses, and the anteroventral orientation of transverse processes indicate the potential for dorsoventral flexion of the trunk and vertebral column in general. Flexion, however, was limited by the anteroposterior width and length of the spinous processes of the lumbar vertebrae.

The sacrum is composed of four vertebrae, the first three fully co-ossified and the fourth with fused pleurapophyses. Robust auricular processes are present on S1 for articulation with ilia of the pelvis. The spinous processes of all sacral vertebrae have massive bases. Presence of a solidly fused sacrum precluded smooth undulatory motion of the body during swimming.

The caudal series is composed of 21 vertebrae (Figures 1C, D, 9). The first 14 caudals have associated hemal arches or chevron bones. Anterior caudals are robust, with substantial spinous processes, metapophyses, and transverse processes. The latter are divided in Ca 4–6. Mid-caudal vertebrae are also large but have more diminutive processes. The size and complexity of vertebrae in the posteriormost part of the tail decrease rapidly following the last chevron-bearing caudal (Figures 1C, D, 9).

The ribs are elongate and moderately curved, with no sign of pachyostosis or osteosclerosis. All but the last few are double-headed. The sternum includes eight elements with a T-shaped manubrium, six sternebrae, and an elongate xiphisternum.

The delicate scapula is longer than wide, with a projecting acromion and shallow glenoid; the coracoid process forms a large tubercle above the glenoid. The scapular spine divides the scapular blade unequally into smaller anterior and larger posterior portions. The humerus is relatively long, flattened, and anteriorly curved. Proximally, the head is ovoid with a narrow bicipital groove. Distally, the humerus is narrow with a deep trochlea for the ulna. Posteriorly, there is a deep olecranon fossa. The convex articulation for the radial head is located lateral to the ulnar trochlea. The medial epicondyle is robust and projecting. Ulna and radius are shorter than the humerus (Figure 7). The ulna is flattened and has a large, blade-shaped olecranon process. The trochlear notch of the ulna projects farther anteriorly, locking into the humeral olecranon fossa during extension. The notch is bifid with a broad articulation distally for the radius. Although the radius and ulna are not coossified, they were fixed in a pronated position. The head of the radius, the shortest forelimb element, has a square contour and saddle-shaped surface. The radial shaft, unlike that of the humerus and ulna, has a subtriangular cross-section. The robust distal end of the radius has a distinct oval, flat articular surface for the carpus.

The carpus is composed of a series of proximal and distal carpals that vary in shape from ovoid to polygonal (Figure 7). Proximal and distal carpal rows are staggered with interlocking articulations. The pisiform projects strongly ventrally, more than required to maintain the aponeurosis of the carpal tunnel, providing a powerful mechanical advantage for the flexor carpi ulnaris. The prominent medial malleolus on the humerus provides the origin for this muscle. The pisiform resembles the fifth metacarpal (Mc-V) in size and morphology. The carpals are transversely arched providing a deep ventral tunnel for flexor tendons. Digits three and four are dominant and metacarpal length decreases in the order of Mc-III, Mc-IV, Mc-II, Mc-V, and Mc-I (Table 1). Large, paired sesamoids are located on the ventral surfaces of the head of each metacarpal. Proximal phalanges are long and relatively thin, as are middle phalanges. Distal phalanges are much shorter and somewhat more robust. The manus is shorter than the pes and has more delicate terminal phalanges.

**Figure 10:**
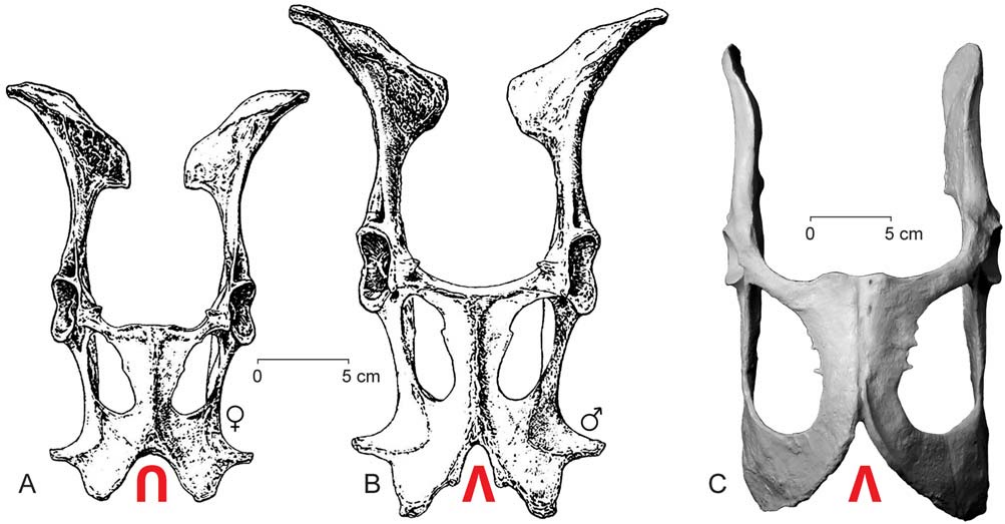
Pelvic morphology in the extant sexually-dimorphic fallow deer *Dama dama* compared to that of male *Maiacetus inuus*. Pelves are viewed ventrally. Sexes differ in the shape of the notch separating inferior rami of left and right pubes. (A)– Female *Dama dama* has the more U-shaped pubic notch labeled in red. (B)– Male *Dama dama* has the more V-shaped pubic notch labeled in red. (C)– Male *Maiacetus inuus*, GSP-UM 3551, has the V-shaped pubic notch labeled in red. Drawings of *Dama dama* are from [29]. Part of the right pubic ramus of GSP-UM 3551 is restored from the left side.

The innominate is anchored to the sacrum. The ilium is short anterposteriorly relative to the length of the pelvis. The round, well buttressed acetabulum is notched with a pit for the ligamentum teres. The obturator foramen is large. The ischium is large, flattened, and extends farther from the acetabulum than does the ilium (Figure 1A). The pubic notch formed by inferior rami of left and right pubic bones is ‘V’ shaped (Figure 10), as expected in a male. The robust femora are short relative to the length of the distal segments of the hind limb. The femoral head is rounded with a deep fovea capitis femora. Robust muscle attachment sites on the femur include prominent greater and lesser trochanters, a deep intertrochanteric fossa, and a distally extended lateral crest for insertion of vastus lateralis. The distal condyles of the femur are offset from one another, which served to rotate the tibia medially during flexion. The relatively long tibia has a robust proximal end and deeply trochleated distal end shaped to receive the astragalus. The relatively long, thin fibula has articular surfaces proximally for the tibia and distally for the calcaneum.

**Figure 11:**
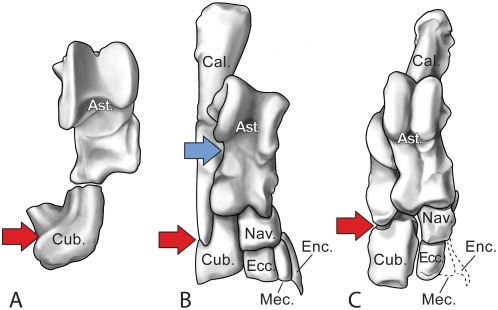
Comparison of right ankle bones of *Artiocetus*, *Maiacetus*, and *Rodhocetus*. (A)–Astragalus and cuboid of *Artiocetus clavis* (GSP-UM 3458, type, reversed). (B)–Ankle of *Maiacetus inuus* (GSP-UM 3551). (C)–Ankle of *Rodhocetus balochistanensis* (GSP-UM 3485, type). Specimens are drawn to approximately the same astragalus+cuboid length, and viewed dorsally. Blue arrow points to distinctive indentation in lateral margin of astragalus of *M. inuus*; red arrows point to the broad and deep, narrow and intermediate, and narrow and shallow indentations in the calcaneum and lateral margins of cuboids. *Abbreviations*: *Ast.*, astragalus; *Cal.*, calcaneum; *Cub.*, cuboid; *Ecc.*, ectocuneiform; *Enc.*, entocuneiform; *Mec.*, mesocuneiform; *Nav.*, navicular.

The ankle, preserved in articulation on both sides in GSP-UM 3551, resembles that of larger-bodied *Rodhocetus balochistanensis* more than that of similar-sized *Artiocetus clavis* (Figure 11). The astragalus is ‘double-pulleyed’ with a trough-shaped trochlea for the tibia and an opposing trochlea for the navicular. Other astragalar facets include a narrow convex articular surface for the cuboid adjacent to the deeply notched surface for the navicular, a small curved surface laterally for articulation with the ectal facet of the calcaneum, a proximodistally elongated convex facet ventrally for long excursion on the calcaneal sustentacular surface, and a small facet adjacent to the cuboid facet for articulation with the calcaneum. On the calcaneum, the tuber is long and robust; the sustentaculum is oval, small, and shelf-like; the ectal facet is convex and confluent with the curved facet for the fibula; and the distal cuboid facet is slightly concave and obliquely oriented. The deeply concave navicular has a proximal keel for articulation with the astragalus, and distal facets for articulation with the ento-, meso-, and ectocuneiform (Figure 11). The cuneiforms are small and subrectangular with simple articular surfaces for the second and third metatarsals (Mt-II and Mt-III). It is possible that a tiny distal extension of the entocuneiform accommodated a very diminutive (but unrecovered) Mt-I. A pair of proximal articular surfaces on the cuboid include a concave medial portion for the astragalus and an obliquely notched, flatter surface for articulation with the distal surface of the calcaneum. Distally, the cuboid has a larger, flat articular surface for Mt-IV and a smaller articular surface for Mt-V.

Pedal digits III and IV are largest. Lengths decrease in the sequence Mt-IV, Mt-III, Mt-V, and Mt-II. Each metatarsal head has paired facets ventrally for articulating sesamoid bones. Proximal and middle phalanges are long and relatively thin. Ungual phalanges are much shorter, ventrally flattened, and ornamented with dorsal tubercles and sulci to accommodate extensor tendons.

## Discussion

### Extraordinary Fossils

The fossils described here include the first association of adult female and fetal whale skeletons, the latter apparently near term and in birth position (GSP-UM 3475a, b) (Figure 2). The adult male skeleton (GSP-UM 3551) found nearby is exceptionally complete (Figure 9). Together these specimens document a new protocetid whale preserving a complete vertebral column and complete fore- and hind limbs. The vertebral formula of *Maiacetus* is similar to that preserved in the other protocetids *Rodhocetus* and *Qaisracetus* (7C-13T-6L-4S) [14], [16], and in addition indicates the presence of 21 caudal vertebrae. As in all living semiaquatic mammals, the limbs are relatively short and pelvic girdles provide a direct connection to the vertebral column for weight-bearing. The ankles are artiodactyl-like, with a double-pulley astragalus, notched cuboid, and curved fibular facet characteristic of artiodactyls [3]. Metapodials are elongated, and the manual and pedal digits were almost certainly webbed.

**Figure 12:**
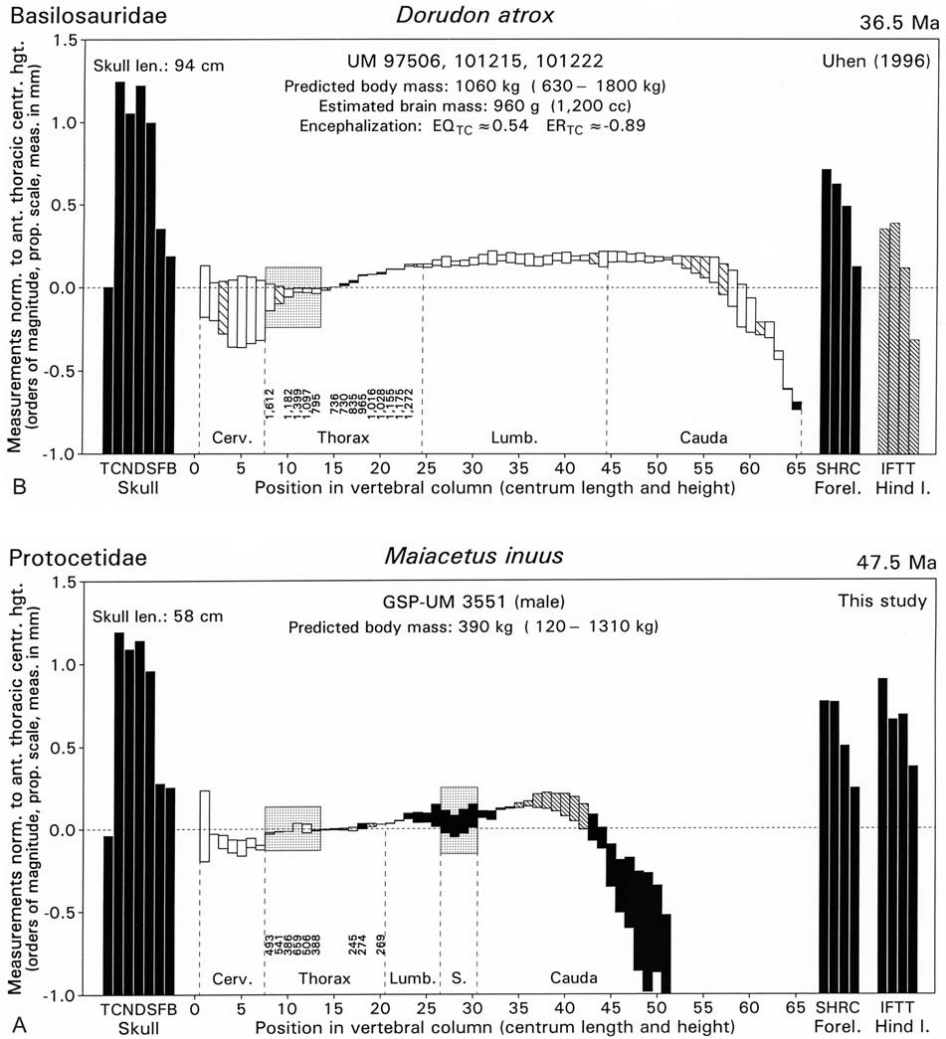
Proportion-adjusted skeletal profiles of middle and late Eocene archaeocete whales *Maiacetus inuus* and *Dorudon atrox*, respectively. (A)—*Maiacetus inuus*, a semiaquatic foot-powered swimmer from the Middle Eocene. (B)—*Dorudon atrox*, a fully-aquatic tail-powered swimmer from the Late Eocene. Baseline is mean length of anterior thoracic vertebrae (stippled box). Sacral vertebrae are enclosed in a second stippled box where sacrals can be identified (e.g., by co-ossification). *Maiacetus* has a profile more like that of mammals capable of supporting their weight on land, whereas *Dorudon* has the profile of a modern whale. Interpretation of profiles and the method of median serial-multiple-regression estimation of body weights is explained in [50]. *Abbreviations*: *TCNDSFB*, longest tooth length, condylobasal cranium length, narial position, dentary length, symphysis position, mandibular foramen height; and bulla length, respectively; *SHRC*, scapula, humerus, radius, and Mc-III lengths, respectively; *IFTT*, innominate, femur, tibia, and Mt-III lengths, respectively.

**Figure 13:**
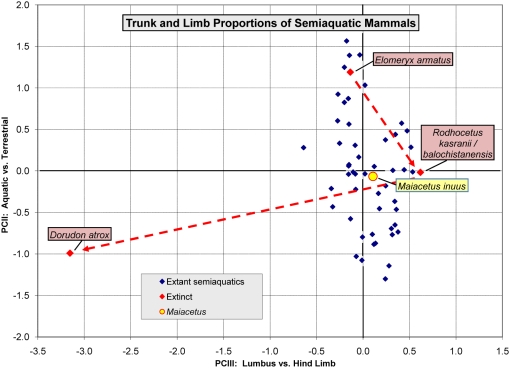
Principal components plot of trunk and limb skeletal proportions for *Maiacetus inuus* and a representative sample of 50 extant semiaquatic mammals. *Maiacetus inuus* is similar to *Rodhocetus* species, but lacks elongation of the manus, hind limb, and pes characteristic of the latter. *Maiacetus* is about equally aquatic (PC-II) to *Rodhocetus* but less specialized as a hind-limb swimmer (PC-III). It falls closest to the giant otter (*Pteronura brasiliensis*) among extant mammals. Background and comparative data for this analysis are documented and explained in [4].

*Maiacetus* has a piscivorous dentition and, like other early protocetids, is interpreted as an amphibious, semiaquatic, foot-powered swimmer that fed in the sea and came ashore to rest, mate, and give birth, as anticipated by Fordyce [5]. While the hind limbs were capable of bearing the weight of the body on land, the proportions of the limbs and the long phalanges of both hands and feet would have limited terrestrial locomotion and prevented *Maiacetus* from traveling any substantial distance from water. *Maiacetus* differs from other early protocetids in having dentaries fused at the mandibular symphysis (Figure 5), ankles of distinct proportions (Figure 11), and limbs slightly different in proportion from those of other early protocetids (Figures 12, 13). These differences suggest that there are feeding and swimming specializations among protocetids that remain to be clarified.

### Birth on Land

Presence of an intact fetal skeleton (GSP-UM 3475b) enables the first unequivocal determination of sex for an archaeocete: GSP-UM 3475a is female. The presence of one fetal skeleton and no trace of a second indicates that birth in *Maiacetus* involved a single calf. Early archaeocetes thus resemble all extant large semiaquatic mammals, which invest their energy in gestation and parenting of a single infant per breeding event [17].

**Figure 14:**
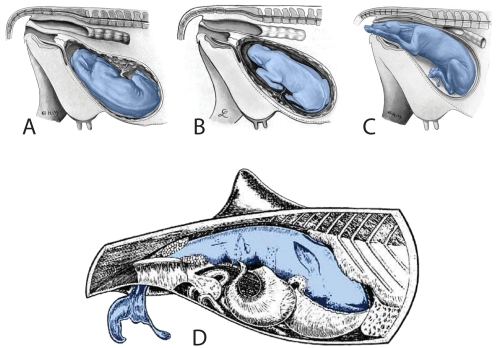
Head versus tail presentation of near- and full-term calves in a domestic cow and harbor porpoise. Domestic cow (*Bos taurus*) calf (blue) in (A) ninth and final month of gestation before ‘turning’ (axially rotating), (B) turned as the birth process begins, and (C) with forelimbs and head partially extruded in the initial stage of birth (from [51], [52]). Harbor porpoise (*Phocaena phocaena*) (D) with full-term calf (blue) with tail partially extruded in the initial stage of birth (from [18]).

The rostrum of the fetal skeleton is pointing opposite that of the mother (Figure 2). Posterior orientation of the fetal head occurs in the initial stage of birth in artiodactyls (Figure 14C). However, given the shorter neck in *Maiacetus*, this may have been the fetal position during all of late gestation. Because the head of the fetal skeleton of *Maiacetus* is not in the mother's pelvic canal, the fetus may have been near term but not full term. Partially formed M1 crowns in the fetal cranium suggest that the fetus was at least near term.

The fetal skeleton is positioned for head-first birth, a universal birthing posture in large-bodied land mammals, but one that is anomalous in fully-aquatic marine mammals [18], [19], [20]. A near-term fetus in an ungulate or whale may rotate about its long axis as it passes through the birth canal, but it cannot turn head-to-tail (Figure 14A–C). Discovery that early whales delivered calves like land mammals indicates that birth in semiaquatic protocetids still took place on land.

Cephalic presentation at birth is generally held to be advantageous on land as it enables a newborn to breath during labor. Caudal presentation at birth, in contrast, is generally held to be advantageous at sea as it may reduce the risk of drowning [18]. Caudal presentation may also hold an advantage in water, because it orients the newborn calf to swim parallel to the mother rather than away from her. This might be important for communication, initiation of nursing, and protection from predators. Cephalic presentation does not incur the same risks on a solid substrate.

Birth at sea was a prerequisite for the first fully-aquatic whales, the Basilosauridae (including *Dorudon*; Figure 1C, D), which evolved from Protocetidae later in the Eocene. Basilosauridae have reduced hind limbs that no longer contact the vertebral column and could not support the weight of the body on land. Hence they could no longer come out of the sea onto land to give birth. With their innominate decoupled from the vertebral column, the birth canal in basilosaurids may have been considerably larger than that in protocetids. This would have permitted birth of larger, more precocial infants, and we would predict that a near-term basilosaurid fetal skeleton, if found intact, would be positioned to be born tail-first as is seen in living whales.

### Precocial Development

The dentition of the fetal skeleton (GSP-UM 3475b) can be mapped in detail (Figure 8). Here partial crowns of the first permanent upper molars (left and right M^1^) are visible posterior to dP^4^ (best exposed on the left side). The main paracone cusp appears almost fully formed, with the crown as a whole being about one-half mineralized. Formation of the crowns of many deciduous teeth, and particularly formation of M^1^, indicates that the fetal skeleton of *Maiacetus* was advanced in development and near full term.

Altricial-to-precocial birth status in mammals is generally represented on a behavioral spectrum scored from I to IV. Altricial I lies at one extreme (‘birth with eyes closed, naked, and nidicolousor nest-dwelling’), and precocial IV at the other (‘birth with eyes open, haired, and nidifugous or nest-fleeing’) [21]. Dental development provides a parallel indication of birth status (Table 4). The state of the dentition at birth is a direct indicator of the ability of a newborn to supplement mother's milk with other foods. Natural selection can separately adjust a newborn's ability to feed itself, sense its environment, regulate its temperature, and move [24], yet there is also an overall association of these aspects of newborn maturity. Table 4 shows that mineralization of the dentition at birth corresponds to the classic categories of neonate maturity. In generalized mammals, crowns of the deciduous dentition appear to be largely mineralized *in utero*. The permanent dentition, however, shows an enormous range, with some mammals showing no mineralization of permanent teeth at all (e.g., a newborn bear cub [22]), and others having all adult crowns fully formed (e.g., newborn guinea pig [23]). While eruption of teeth through the gums in living mammals is informative, the clearest association with precocial categories II–IV is some degree of mineralization of the permanent dentition, beginning with the first molar and in some cases extending to mineralization of later developing teeth.

**Table 4:** Developmental maturity categories of Langer [21] and mineralization of first molar M^1^ and other permanent teeth in newborn terrestrial mammals compared to the early whale *Maiacetus inuus*.

Species	Mineralization of the permanent dentition at birth	Reference
**Category I. Altricial: eyes closed, naked, nidicolous or ‘nest-dwelling’**		
Gray short-tail opossum, *Monodelphis domestica*	None	[33]
Brown bear, *Ursus arctos*	None	[22]
Indian mongoose, *Herpestes auropunctatus*	None	[34]
Ferret, *Mustela putorious*	None	[35]
Domestic dog, *Canis familiaris*	Trace	[36]
**Category II. Precocial: eyes open, haired, nidicolous or ‘nest-dwelling’**		
Domestic pig, *Sus scrofa*	None	[37]
Miniature pig, *Sus scrofa*	M1 cusp tips beginning to mineralize	[38]
Collared peccary, *Tayassu tajacu*	M1 cusp tips formed	[39]
**Category III. Precocial: eyes open, haired, transported (largely primates)**		
Human, *Homo sapiens*	M1 cusp tips beginning to mineralize	[40]
Great apes, *Pan*, *Gorilla*, and *Pongo* spp.	M1 cusp tips beginning to mineralize	[40]
Gibbon, *Hylobates* sp.	M1 cusp tips beginning to mineralize	[40]
Rhesus macaque, *Macaca mulaltta*	M1 cusp tips formed	[41]
Mongoose lemur, *Eulemur mongoz*	M1 cusp tips formed	[42]
Coquerel's sifaka, *Propithecus verreauxi*	M1 crown almost complete, M2 near coalescence, M3 trace	[42]
Tarsier, *Tarsius bancanus*	M1 ‘well differentiated’ (some deciduous teeth resorbed *in utero*)	[43]
**Category IV. Precocial: eyes open, haired, nidifugous or ‘nest-fleeing’**		
American tapir, *Tapirus cf. T. bairdii*	M1 anterior cusps connected	[44]
Domestic sheep, *Ovis aries*	M1 cusps connected; crown one-quarter formed	[45]
***Maiacetus inuus*** 1	**M1 crown one-half formed**	this study
Fallow deer, *Dama dama*	M1 crown one-half formed	[46]
Pygmy hippo, *Hexaprotodon liberensis*	M1? (many deciduous teeth erupted at birth; large M1 crypt)	[27]
Giraffe, *Giraffa camelopardalis*	M1 crown complete (all deciduous teeth erupted at birth)	[47]
Fur seal, *Callorhinus ursinus*	M1-2 crowns complete (deciduous teeth shed *in utero*)	[48]
Guinea pig, *Cavia porcellus*	All permanent teeth erupted; roots incomplete	[23]

Living whales are not included because none have deciduous teeth and many have few or no teeth. Because early whales evolved from artiodactyls [3], *Ovis*, *Dama*, *Hexaprotodon*, and *Giraffa* are better analogs than primates.

1 Presence of mineralization of the crown of M^1^ in the fetal skull of *Maiacetus inuus* indicates precocial development and the probability that newborns were open-eyed, hairy and nidifugous.

The fetal skeleton of *Maiacetus* described here shows development of the deciduous dentition to have been well underway, with partial mineralization of upper first permanent molars *in utero*. The fetal dentition of *Maiacetus* is as developed as that of a newborn fallow deer (Table 4). Thus *Maiacetus* was clearly a precocial mammal.

Precociality involves a high degree of maternal investment starting from conception and extending through relatively long periods of gestation and lactation to nutritional independence of the calf. Marine mammals living today are all precocial, and energy is channeled to early growth [25]. Semiaquatic young must be able to move soon after birth to avoid predators on land and in the sea, and fully aquatic young must be able to swim to keep pace with their mothers [17]. Larger newborn body size is also an important component of precociality because it reduces the newborn's surface-to-volume ratio and enhances its ability to maintain its body temperature on exposed shorelines and in the water.

Artiodactyls, the group ancestral to whales [3], give birth to young that range from slightly precocial (pigs) to highly precocial (sheep, deer, giraffe). Whales probably evolved from anthracobunid artiodactyls, a stem group that may be closely related to Hippopotamidae [26]. Hippopotamid young lie at the precocial end of the birth spectrum [27], [28], and it is reasonable to infer that precociality was primitive for whales and their closest artiodactyl ancestors. Precociality may be a key life history trait that enabled the earliest whales to navigate the land-sea transition.

### Sexual Dimorphism

The type specimen of *Maiacetus inuus*, GSP-UM 3475a, has a fetus *in utero* and is clearly female. We interpret the referred adult skeleton of *Maiacetus* (GSP-UM 3551) as male because it contains no fetus, averages 12% larger in linear measurements than the known female, and has canine teeth that are 20% larger (Tables 1, 2). A 12% difference in linear measurements of male and female *Maiacetus* yields an expected weight difference of about 39% (Table 1). Relatively large canines are commonly found in male mammals that use these teeth in threat displays and fighting. In addition, the pelvis of GSP-UM 3551 is well preserved and has a V-shaped pubic notch, formed by the inferior rami of the left and right pubes, which is characteristic of males (Figure 10). The notch is broader and U-shaped in females that give birth to a single large fetus, such as the fallow deer *Dama dama* [29].

**Figure 15:**
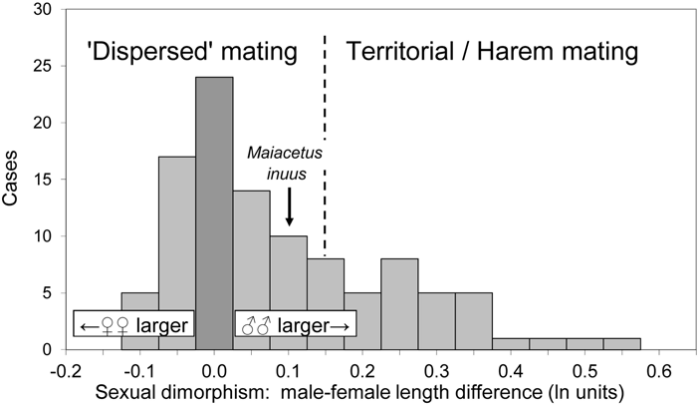
Mating systems and sexual dimorphism in marine mammals. Sexual dimorphism of males and females is quantified for 105 species of cetaceans, pinnipeds, sirenians, and the sea otter, expressed as male-minus-female length in natural log units [17], [30]. Territorial and harem systems that involve intense male-male competition and spatial aggregation of females have male-female dimorphism greater than about 0.15 (dashed line) [31]. *Maiacetus inuus* falls in the group of ‘dispersed’ mating systems with limited aggregation and limited male control of mating. Both cetaceans and pinnipeds span the entire range shown here, although mating systems are known for relatively few species [32].

Dimorphism of body size in marine mammals ranges from females that are larger than males (Hawaiian monk seal and Gervais' beaked whale at the extreme) to males that are much larger than females (sperm whale, southern elephant seal, and Hooker's sea lion at the extreme) [17], [30]. Here we quantify the difference in terms of body length (Figure 15). The mating system is not known for all marine mammals, but when known, a male-female length difference greater than about 16% (0.15 on a natural log scale) indicates a territorial or harem mating system [31]. Intense male-male competition is associated with marked sexual dimorphism and often spatial aggregation of females related to the accessibility of food and shelter. A male-female length difference less than 16% corresponds to a more generalized ‘aquatic,’ or ‘dispersed’, mating system, in which males have less opportunity to guard and monopolize females.

The male-female length difference of 12% (0.11 on a natural log scale) in *Maiacetus* indicates moderate sexual dimorphism and probably limited male-male competition for mates. Limited opportunity to monopolize mates suggests in turn that food and shelter were dispersed in protocetid habitats. This is corroborated by the geographically extensive but environmentally uniform shallow marine deposits of the Habib Rahi Formation, which were deposited on a broad, shallow marine shelf that would have provided little spatial aggregation of food or shelter.

Much remains to be learned about mating systems in cetaceans because their behavior is so difficult to study [32]. At present, body size dimorphism is itself sometimes used as a measure of ‘contest competition’ between males [17]. *Maiacetus* suggests that sexual dimorphism appeared early in cetacean evolution. Males were larger than females, but the difference was modest on the scale of dimorphism observed in marine mammals today.

### Conclusions

We describe exceptional specimens of a new early middle Eocene (47.5 Ma) protocetid whale, *Maiacetus inuus*, that include an adult female skeleton, a near term fetal skeleton, and an adult male skeleton. *Maiacetus* differs in size and proportions from younger, more derived basilosaurids such as *Dorudon atrox* (Figures 1, 12, 13), but it is close in size and similar in proportions to a composite skeleton of *Rodhocetus balochistanensis/kasranii* (Figure 13). *Maiacetus* retains many characteristics of its terrestrial forebears, including shearing cheek teeth with protocones on upper molars, a vertebral formula very close to that of primitive artiodactyls, a forelimb retaining mobile digits, a pelvis and hindlimb anchored on the vertebral column; and a double-pulley astragalus within the ankle. The feet of *Maiacetus* are not as elongated as those of *Rodhocetus*, indicating that *Maiacetus* may have been a slightly less specialized foot-powered swimmer (Figure 13).

Preservation of an intact near-term fetal skull and partial skeleton indicates that birth in early archaeocetes involved a single calf that was born head-first as in land mammals, not tail-first as in living whales. This, in turn, indicates that birth almost certainly took place on land during this phase of early whale evolution. The presence of partially mineralized permanent first molars in the fetal skull indicates precocial development, which may have been a key life history trait in early whales facilitating the transition from land to sea. Sexual dimorphism in body and canine size are moderate, suggesting limited competition among males for mates.
